# Assessment of the Association of Leadership Behaviors of Supervising Physicians With Personal-Organizational Values Alignment Among Staff Physicians

**DOI:** 10.1001/jamanetworkopen.2020.35622

**Published:** 2021-02-09

**Authors:** Tait D. Shanafelt, Hanhan Wang, Mary Leonard, Mary Hawn, Quinn McKenna, Rick Majzun, Lloyd Minor, Mickey Trockel

**Affiliations:** 1 Department of Medicine, Stanford University School of Medicine, Stanford, California; 2 WellMD Center, Stanford University School of Medicine, Stanford, California; 3 Department of Pediatrics, Stanford University School of Medicine, Stanford, California; 4 Department of Surgery, Stanford University School of Medicine, Stanford, California; 5 Stanford Health Care, Stanford Medicine, Stanford, California; 6 Stanford Children's Health/Lucile Packard Children's Hospital, Stanford Medicine, Stanford, California; 7 Department of Otolaryngology, Stanford University School of Medicine, Stanford, California; 8 Department of Psychiatry and Behavioral Sciences, Stanford University School of Medicine, Stanford, California

## Abstract

**Importance:**

Although misalignment of values between physicians and their organization is associated with increased risk of burnout, actionable organizational factors that contribute to perceived values alignment are poorly understood.

**Objective:**

To evaluate the association between the leadership behaviors of immediate supervisors and physicians’ perception of personal-organizational values alignment.

**Design, Setting, and Participants:**

This survey study of faculty physicians and physician leaders at Stanford University School of Medicine was conducted from April 1 to May 13, 2019. The survey included assessments of perceived personal-organizational values alignment, professional fulfillment, and burnout. Physicians also evaluated the leadership behaviors of their immediate supervisor (eg, division chief) using a standardized assessment. Data analysis was performed from May to December 2020.

**Main Outcomes and Measures:**

Association between mean leadership behavior score (range, 0-10) of each supervisor and the mean personal-organizational values alignment scores (range, 0-12) for the physicians in their work unit.

**Results:**

Of 1924 physicians eligible to participate, 1285 (67%) returned surveys. Among these, 651 (51%) were women and 729 (57%) were aged 40 years or older. Among the 117 physician leaders evaluated, 66 (56%) had their leadership behavior independently evaluated by at least 5 physicians and were included in analyses. The mean (SD) personal-organizational values alignment score on the 0 to 12 scale was 6.19 (3.21). As the proportion of work effort devoted to clinical care increased, values alignment scores decreased. Personal-organizational values alignment scores demonstrated an inverse correlation with burnout (*r* = −0.39; *P* < .001) and a positive correlation with professional fulfillment (*r* = 0.52; *P* < .001). The aggregate leader behavior score of the 66 leaders evaluated correlated with the mean values alignment score for physicians in their work unit (*r* = 0.53; *P* < .001). Aggregate leader behavior score was associated with 21.6% of the variation in personal-organizational values alignment scores between work units. After adjusting for age, gender, academic rank, work hours, physician-leader gender concordance, and time devoted to clinical care, each 1-point increase in leadership score of immediate supervisor was associated with a 0.56-point (95% CI, 0.46-0.66; *P* < .001) increase in personal-organizational values alignment score.

**Conclusions and Relevance:**

This survey study’s results suggest that physicians experience their organization through the prism of their work unit leader. Organizational efforts to improve values alignment should attend to the development of first-line physician leaders.

## Key Points

**Question:**

Is physicians’ perception of alignment between personal and organizational values associated with their evaluation of the leadership behaviors of their immediate supervisors?

**Findings:**

In this survey study of 1285 physicians and physician leaders, each 1-point increase in the aggregate leadership behavior score of physicians’ immediate supervisor was associated with a 0.65-point increase in the personal-organizational values alignment score for the physicians in their work unit. The aggregate leader behavior score of each leader as assessed by all physicians they lead explained 21.6% of the variation in personal-organizational values alignment scores between work units.

**Meaning:**

The leadership behavior of each physician’s immediate supervisor was associated with the variation in physicians’ perception of values alignment with their organization overall.

## Teaser

This survey study examines whether there is an association between physicians’ perceptions of their immediate supervisor’s behaviors and their views on how their personal values align with those of their organization.

## Introduction

Physicians increasingly are employed by large organizations.^1^ This arrangement works best when physicians and the organizations in which they work have a shared sense of mission and purpose.^2,3,4^ It would be intuitive to infer that such alignment of values would come naturally. Fundamentally, health care organizations should exist to provide high-quality medical care for the patients in their community, which is a mission that aligns well with the professional values of physicians.

Unfortunately, experience suggests such alignment does not come easily.^5,6,7,8,9,10,11^ Physicians often feel that their organizations view them as a commodity or unit of production.^5,6,12^ They frequently project that administrative leaders care more about payer mix, profitable service lines, and net operating income than providing compassionate, expert, and individualized care for the patients served.^8,12^ This perception is promulgated by organizational reliance on financial incentives that focus on relative value unit generation (or throughput) as a strategy to increase physician productivity.^13^ At academic medical centers, many physicians believe that their organization values research activity more than clinical care.^7,14,15^ Collectively, these factors often result in physicians feeling as if their institution views them as a replaceable part rather than a professional.^5,6^ Although such misalignment may stem from fundamental differences in purpose, strategy, and goals, evidence suggests that this is an oversimplification.^16^

Ultimately, the perceived misalignment of values between physicians and their organization breeds distrust, division, and reciprocal scapegoating (physicians blame administrators, administrators blame physicians), which undermine the ability of the organization to achieve its mission.^16^ Although perceived misalignment has been well chronicled, the array of factors that contribute to it are poorly understood. Perhaps more importantly, there is a need to identify specific, targetable interventions that senior leaders and health care organizations can focus on to improve perceived alignment. In the present study, we (1) assessed physicians’ perception of alignment between their personal values and the values of their organization and (2) evaluated the association of the leadership behaviors of their immediate supervisor with perceived values alignment with the organization overall.

## Methods

### Participants and Survey Measures

As previously reported,^17^ Stanford Medicine conducted a survey of its clinical faculty and affiliated physicians in the spring of 2019 to help guide organizational efforts to improve professional fulfillment. The current analysis focused on the 1924 faculty physicians invited to participate in the online survey between April 1, 2019, and May 13, 2019. Medical staff faculty, hereafter referred to as *physicians*, are overwhelmingly physicians but also include a limited number of other PhD-level clinicians (eg, psychologists). Participation in the survey was voluntary. The response rate was determined by the American Association for Public Opinion Research (AAPOR) reporting guideline for studies of internet surveys of named persons.^18^ This study was reviewed by the Stanford University institutional review board and deemed exempt because it involved retrospective analysis of administratively collected data using a completely anonymized data set.

The survey included questions exploring basic demographic characteristics (age, gender) as well as professional characteristics (faculty track, academic rank, percentage work effort dedicated to clinical work, and mean weekly work hours). Gender concordance between a physician and their leader was also determined and recorded for each physician (concordant: male leader and male physician or female leader and female physician; not concordant: male leader and female physician or female leader and male physician).

### Evaluation of Professional Fulfillment and Burnout

The Stanford Professional Fulfillment Index is a well-validated tool to assess professional fulfillment and burnout that is used by organizations across the US and the world.^19,20^ The professional fulfillment domain includes 6 items, whereas the burnout domain includes 10 items (4 items assessing work exhaustion and 6 items assessing interpersonal disengagement). All items are scored on a 5-point Likert scale with options ranging from not at all to extremely for the burnout items and not at all true to completely true for items about professional fulfillment. Aggregate burnout and professional fulfillment scores were determined using the published approach.^19^

### Leadership Behaviors

Each participant was asked to evaluate the leadership behavior of their immediate supervisor by name. Participants began by selecting the name of their supervisor from a dropdown menu that listed the names of their department chair, division chief, and medical directors. These individuals were then evaluated using the Mayo Clinic Participatory Management Leadership Index (eTable 1 in the Supplement).^21^ This instrument was designed to evaluate dimensions of leadership that drive engagement and discretionary effort among team members. The original 12-item instrument was subsequently revised to a 9-item instrument as previously reported.^17^ Each item is scored on a 5-point Likert scale (1 = strongly agree; 5 = strongly agree) and scores are summed to yield a total score. The aggregate leadership behavior score for each leader is calculated by the composite evaluations of all responding physicians they supervise.

### Personal-Organizational Values Alignment

Physicians’ perspectives of the extent to which their personal values aligned well with the values of their institutions was assessed with the 3-item Stanford Values Alignment scale. This instrument was developed through a multistep process by one of the authors (M.T.). The initial scale was refined with feedback from a group of approximately 30 physicians interested in physician wellness prior to initial pilot testing. A preliminary set of 6 items was subsequently tested in a sample of more than 600 physicians in 2016. The scale was then shortened to 3 items found to render good internal consistency, the expected association with professional fulfillment, and inverse correlation with burnout.^22^ The 3 items are: (1) *My input is valued in important administrative decisions*, (2) *Our organizational goals and values fit well with my goals and values, *and (3)* Administration values my clinical work*. Items are scored on a 5-point Likert scale with options ranging from not at all true (score = 0) to completely true (score = 4). Aggregate scores are determined by summing the 0 to 4 score for each of the individual items to yield a total score ranging from 0 to 12. Subsequent to the 2016 study, the instrument has been used by a large number of health care organizations across the US.

To evaluate whether any of the 3 values alignment items assessed themes similar to the construct of the 9 items in the Mayo Clinic Participatory Management Leadership Index, we conducted a principal component analysis with Oblimin rotation and Kaiser normalization to determine underlying patterns between these 12 items (the 3 items from the Values Alignment Scale and 9 items from the Leadership Scale). Two components emerged from these 12 items: all 3 values alignment items clustered as 1 component, and all 9 of the leadership items clustered as the other component, suggesting these domains are distinct constructs (eTable 2 in the Supplement).

### Statistical Analysis

All statistical analyses were performed using R statistical software version 3.6.0 (R Core Team, 2019) from May to December 2020. Statistical significance was set at 2-tailed *P* < .05. All items in the survey were scored using the published approach.^19^ Burnout, professional fulfillment, and the Participatory Management Leadership index were scaled to 0 to 10 to simplify the interpretation of results. Descriptive summary statistics were used to characterize physician participants. Values Alignment scores among different age, gender, faculty track, academic rank, work hours, and percentage clinical time categories were compared using one-way analysis of variation (ANOVA). Pearson correlation coefficients were used to assess the association between each physician’s personal-organizational values alignment score and their professional fulfillment and burnout scores. Pearson correlation was also used to examine the correlation between each supervisor’s aggregate leadership score and the mean personal-organizational values alignment score of physicians reporting to them. A random-effects ANOVA—to account for the nested data structure of multiple physicians reporting to individual leaders—was specified with leadership score as the dependent variable and with a random effect for leader (eAppendix in the Supplement). Mixed-effects models were specified to test the associations between a physician’s evaluation of their immediate supervisor and their values alignment score, with and without adjustment for gender, age, faculty track, academic rank, work hours, physician-leader gender concordance, and percentage clinical time. Ordinary least squares regression was used to estimate the portion of variance in physicians’ values alignment score attributable to their evaluation of their supervisors.

## Results

As previously reported,^17^ of the 1924 physicians invited to participate, 1285 (response rate, 67%) completed surveys. Among these, 651 (51%) were women and 729 (57%) were aged 40 years or older. Among the 117 physician leaders evaluated, 66 (58%) had their leadership behavior independently evaluated by at least 5 physicians from their work unit and were included in analyses. A total of 871 leadership evaluations were received for these 66 leaders (median [interquartile range], 11 [8-15] evaluations per leader). Among these, 868 (99.7%) were from physicians who also responded to the personal-organizational values alignment questions. The demographic characteristics of the 868 participants are shown in Table 1. Participants were roughly evenly distributed by gender (449 women [51.7%]) and a majority (521 respondents [60.0%]) were aged 40 years or older. With respect to faculty track, 536 physicians (61.8%) were in a clinician educator track, whereas 312 physicians (35.9%) were either in a physician investigator or a biomedical scientist track. The amount of time dedicated to clinical work varied, but 636 participants (73.3%) devoted more than 40% of their professional work effort to clinical care.

**Table 1.** Demographic Characteristics of Physicians Who Evaluated Their Immediate Supervisor

Variable	Physicians, No. (%) (N = 868)
Age group, y	
30-39	239 (27.5)
40-49	240 (27.6)
50-59	150 (17.3)
≥60	131 (15.1)
Missing	108 (12.4)
Gender	
Male	389 (44.8)
Female	449 (51.7)
Missing	30 (3.5)
Work hours	
Mean (SD)	55.74 (14.85)
Missing	3 (0.3)
Time worked weekly, h	
<40	148 (17.1)
41-50	214 (24.7)
51-60	290 (33.4)
61-70	125 (14.4)
>70	88 (10.1)
Missing	3 (0.3)
Faculty track	
Clinician educator	536 (61.8)
Physician investigator or biomedical scientist	312 (35.9)
Other	19 (2.2)
Missing	1 (0.1)
Academic rank	
Instructor	65 (7.5)
Assistant professor	332 (38.2)
Associate professor	200 (23.0)
Professor	268 (30.9)
Missing	3 (0.3)
Percentage of work effort dedicated to clinical care	
1-20	90 (10.4)
21-40	133 (15.3)
41-60	153 (17.6)
61-80	240 (27.6)
81-100	243 (28.0)
Missing	9 (1.0)
Personal–organizational values alignment score^a^	
Mean (SD)	6.19 (3.21)
Missing	3 (0.3)
Burnout score^b^	
Mean (SD)	2.83 (1.84)
Missing	3 (0.3)
Professional fulfillment score, mean (SD)^b^	6.51 (1.99)

^a^ The score range is 0 to 12.

^b^ The score range is 0 to 10.

**Figure 1.**
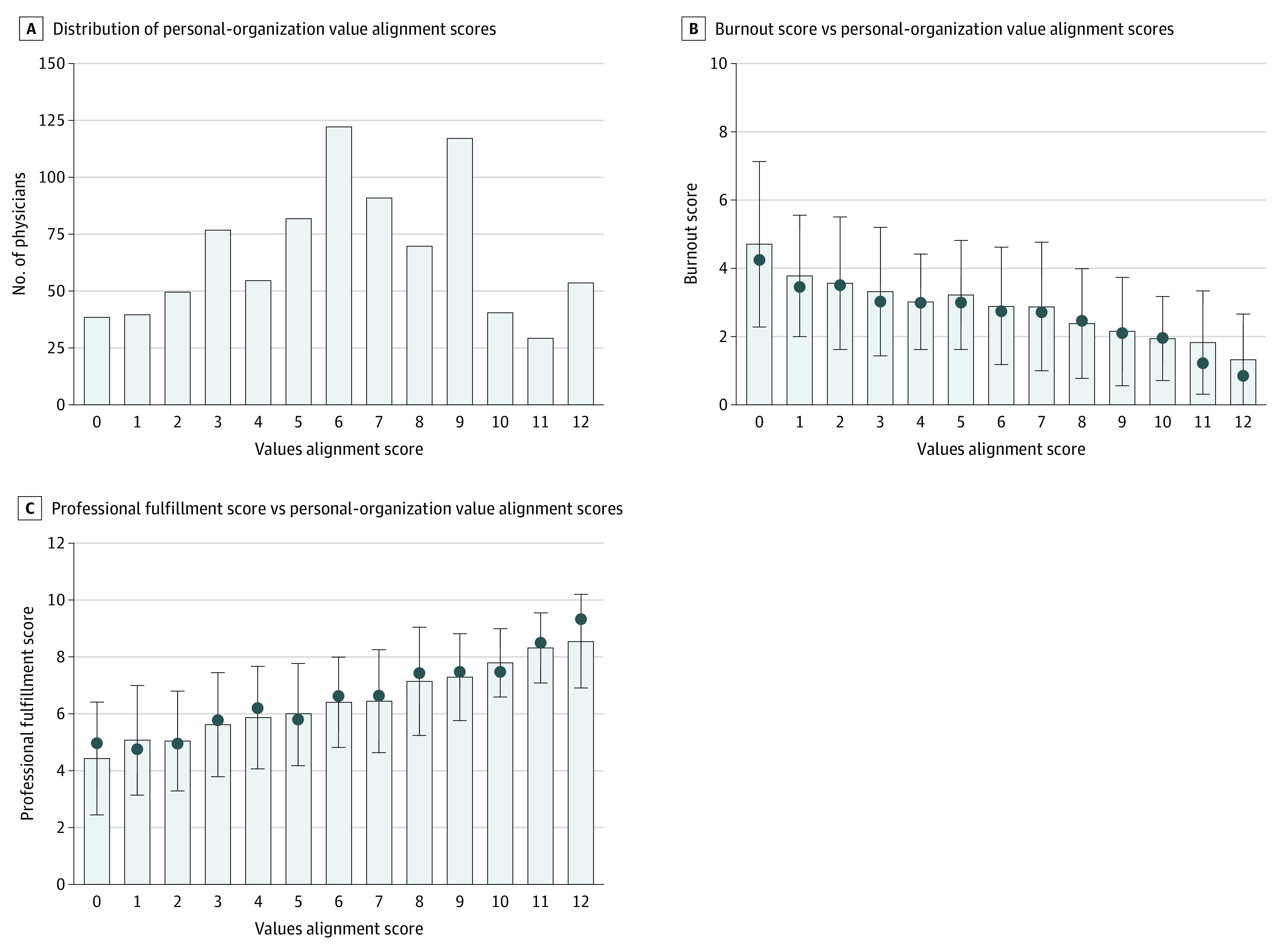
Association of Personal-Organizational Values Alignment Scores With Occupational Burnout and Professional Fulfillment In panels B and C, circles denote medians, and error bars denote 95% CIs.

Responses to the individual items on personal-organizational values alignment are shown in eTable 3 in the Supplement. The scale demonstrated high internal consistency (Chronbach α = 0.87). In aggregate, the mean (SD) personal-organizational values alignment score on the 0 to 12 scale was 6.19 (3.21) (Figure 1A). Variation in values alignment scores by age, gender, work hours, faculty track, academic rank, and amount of time dedicated to clinical work is shown in Table 2. Women physicians reported lower mean (SD) perceived values alignment with their organization than their male colleagues (5.78 [3.08] vs 6.84 [3.24]; *P* < .001). Having a higher proportion of work effort devoted to clinical care was associated with lower values alignment scores (mean [SD], 6.91 [3.22] for physicians with ≤40% effort devoted to clinical care vs 5.97 [3.15] for physicians with >40% effort devoted to clinical care; *P* < .001). No significant association between values alignment score and age, academic rank, faculty track, or work hours was observed. In a multivariable linear mixed model that adjusted for age, faculty track, academic rank, work hours, physician-leader gender concordance, and amount of time devoted to clinical work, being female was associated with having a 1.25-point lower values alignment score (95% CI, −1.80 to −0.70 points; *P* < .001) (eTable 4 in the Supplement).

**Table 2.** Variation in Personal-Organizational Values Alignment Score

Variable	Values alignment score, mean (SD)^a^	*P* value
Age group, y		
30-39	6.18 (3.07)	.80
40-49	6.18 (3.12)	
50-59	6.33 (3.24)	
≥60	6.49 (3.33)	
Missing	5.62 (3.49)	NA
Gender		
Male	6.84 (3.24)	<.001
Female	5.78 (3.08)	
Missing	3.80 (2.77)	NA
Time worked weekly, h		
<40	6.43 (3.21)	.10
41-50	6.22 (3.13)	
51-60	6.33 (3.13)	
61-70	6.14 (3.14)	
>70	5.33 (3.62)	
Missing	5.00 (5.20)	NA
Faculty track		
Clinician educator	6.02 (3.10)	.12
Physician investigator or biomedical scientist	6.49 (3.34)	
Other	6.37 (3.70)	
Academic rank		
Instructor	6.25 (3.02)	.25
Assistant professor	5.93 (3.12)	
Associate professor	6.29 (3.08)	
Professor	6.44 (3.40)	
Missing	4.00 (6.93)	NA
Percentage of work effort dedicated to clinical care		
1-20	6.96 (3.18)	.004
21-40	6.87 (3.25)	
41-60	5.85 (3.12)	
61-80	6.13 (3.25)	
81-100	5.88 (3.09)	
Missing	4.00 (4.15)	NA

Abbreviation: NA, not applicable.

^a^ The score range is 0 to 12.

As a continuous variable, values alignment score demonstrated a significant correlation with both occupational burnout (*r* = −0.39; *P* < .001) and professional fulfillment (*r* = 0.52; *P* < .001). The association between values alignment score and burnout is shown in Figure 1B. Having a higher personal-organizational values alignment score was associated with lower burnout scores. In an inverse manner, having a higher personal-organizational values alignment was associated with higher professional fulfillment scores (Figure 1C).

### Leadership Behavior of Immediate Supervisor and Personal-Organizational Values Alignment in the Work Unit

**Figure 2.**
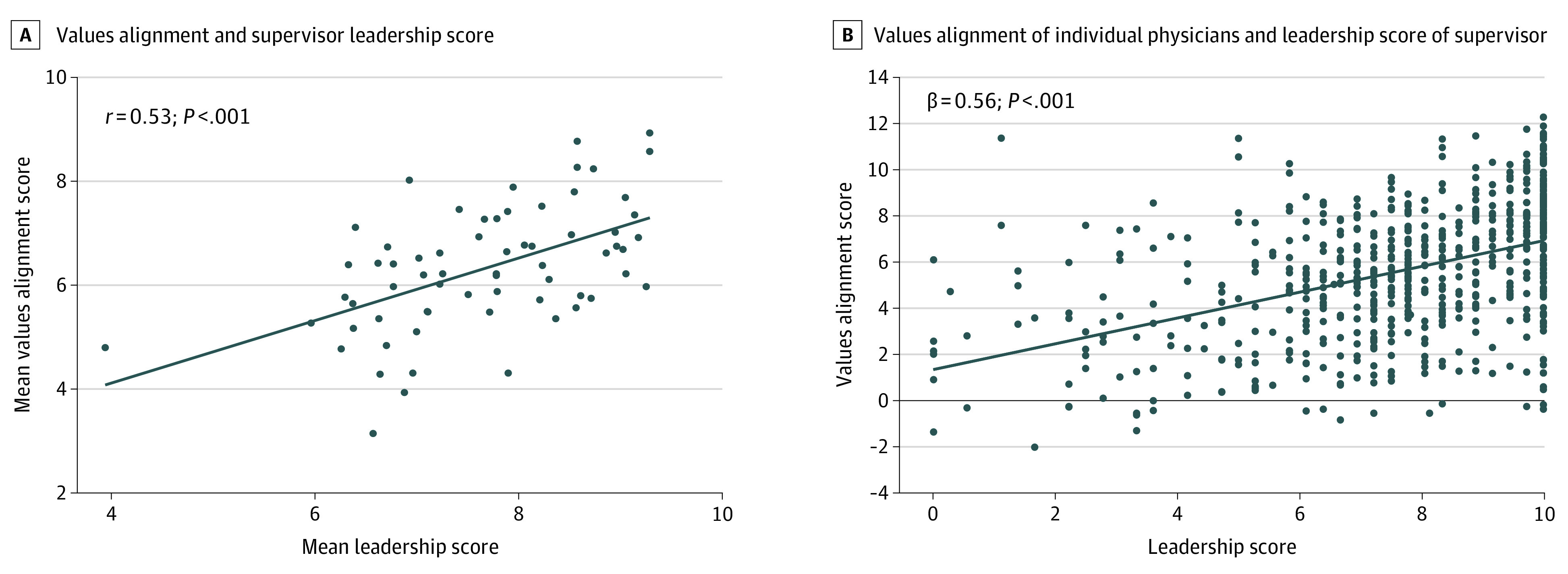
Association of Personal-Organizational Values Alignment With Aggregate Leadership Score of Work Unit Supervisor A, Mean values alignment score of individuals in work unit on the y-axis with the mean aggregate leadership score of that work unit’s supervisor on the x-axis. B, Partial residual plot showing values alignment score of individual physician on the y-axis and leadership score of immediate work unit supervisor on the x-axis after controlling for age, gender, faculty track, academic rank, physician-leader gender concordance, percentage clinical work, and work hours.

Leadership behavior scores for the 66 physician leaders in the analysis ranged from 0 to 10 with a mean (SD) score of 7.57 (2.31). The leadership scale demonstrated high internal consistency (Chronbach α = 0.96). The association between the aggregate leader behavior score (the score for each of the 66 leaders based on mean rating from all the physicians they supervise) and the aggregate personal-organizational values alignment score for all participating individuals in their unit is shown in Figure 2A. Mean aggregate leader behavior score was correlated with perceived values alignment with the organization for physicians in that leader’s work unit (*r* = 0.53; *P* < .001). In this univariable model, physicians’ evaluation of their leader’s behavior was associated with 21.6% of the variation in values alignment score. Univariable linear mixed models clustered by each rated leader indicated that each 1-point increase in leadership score was associated with a 0.65-point increase in personal-organizational values alignment score (95% CI, 0.57-0.73; *P* < .001). After adjusting for age, gender, faculty track, academic rank, work hours, physician-leader gender concordance, and amount of time devoted to clinical care, each 1-point increase in work unit leader leadership score was associated with a 0.56-point (95% CI, 0.46-0.66; *P* < .001) increase in physicians personal-organization values alignment score (Figure 2B). Although a significant association with personal-organizational values alignment score was observed according to whether gender was concordant between a physician and their leader (gender concordant vs not concordant: mean [SD], 6.51 [3.27] vs 6.01 [3.11]; *P* = .04) on univariable analysis, gender concordance between a physician and their leader was not associated with values alignment in the adjusted model.

## Discussion

A shared sense of purpose and alignment of values with health care professionals is critical for health care organizations to achieve their mission. In the present study, we observed an association between the leadership behavior score of physicians’ immediate supervisor and physicians’ perception of values alignment with their organization overall. Our results suggested that 21.6% of the variation in physicians’ perceived values alignment with the organization could be explained by the behavior of the work unit leader. This association persisted after adjusting for age, gender, work hours, faculty track, physician-leader gender concordance, and amount of time devoted to clinical work. These findings suggest the magnitude to which people experience and judge their organization through their work unit leader. Importantly, they also provide a potential target for institutional efforts to advance a sense of shared purpose and values alignment through development of first-line leaders.

Personal-organizational values alignment was also positively associated with professional fulfillment and inversely correlated with occupational burnout. These findings are consistent with previous studies demonstrating an association between values alignment and physician well-being.^8,10,23,24^ Previous studies also provide evidence that perceived values alignment between physicians and their leaders is associated with objective measures of the quality of care provided by those physicians.^8^ These studies further demonstrated that clinics with better values alignment at baseline were more likely to report improvements in the practice environment over time.^25^ Women physicians in our study reported lower perceived values alignment with the organization than their male colleagues, a difference that persisted after adjusting for age, work hours, physician-leader gender concordance, faculty track, academic rank, and the amount of time dedicated to clinical work. This finding suggests that perceived differences in personal-organizational values alignment is yet another factor that may be associated with professional fulfillment of physicians differently on the basis of gender.^26,27,28,29,30,31^ It is also notable that, in this sample of academic physicians, values alignment decreased as the amount of professional work effort devoted to clinical care increased. This observation is consistent with previous studies^7,14,15^ suggesting that physicians practicing at medical centers engaged in research often believe their organization values research more highly than clinical care.

Practicing medicine within a large organization represents a departure from many of the practice models of the past.^1^ This structure often decreases physicians' sense of autonomy, flexibility, and control over their work.^8,32,33^ The size and complexity of today’s health care organizations also makes it difficult for physicians to understand the rationale for many decisions and how they align with the needs of their patients and the altruistic values of the profession. The job of the work unit leader is to help translate the organizational strategy (the *why*) to the work of the local unit and engage the members of their team to determine how to achieve the objectives (the *what* and *how*).^34^ Cultivating engagement, seeking input, and building consensus among a group of physicians with strong opinions and who care deeply about their work and practice environment is not easy.^4,35^ Doing so is particularly challenging for first-line leaders who typically have limited experience with these skills and are typically not provided training and support to develop them. Helping set the vision with the team and giving team members the opportunity to help develop the plan on how to achieve it is a critical skill for first-line leaders. It is also essential that these leaders help transmit appreciation and gratitude for the work that physicians do.

Our findings add to the growing body of literature on the critical role of leadership in the professional well-being of today’s physicians. Evidence suggests that the behavior of immediate supervisors is associated with physician burnout and explains a large proportion of their professional fulfillment.^21^ Across the broad universe of leadership skills, keeping people informed, seeking their input in decision-making, understanding their personal passions, facilitating career development, and recognizing their accomplishments appear to be some of the most critical to the well-being of team members. Recent evidence also indicates that leaders’ own well-being impacts their independently rated effectiveness in these domains.^17^ Organizations can help leaders develop these skills through regular assessment, feedback, training programs, and professional coaching. With respect to the leadership skills associated with the well-being of team members, training and application coaching related to emotional intelligence, creating psychologic safety, humble inquiry, understanding intrinsic motivators, building consensus, appreciative inquiry, improvement methods, and leading through influence may be particularly helpful.^36,37^

### Limitations

Our study has a number of important limitations. First, it is based on analysis of physicians at a single academic medical center. Although it seems unlikely that the association of local leaders’ behavior with physicians’ perception of values alignment with their organization is an institution-specific effect, the degree of perceived values alignment likely varies across institutions. It is also likely that some findings, such as the association between time dedicated to clinical work and values alignment scores, could be specific to academic medical centers. The findings may also be less relevant for physicians without a clear supervisor working in loosely organized medical groups. Second, our data are cross-sectional and we are unable to determine causality or the potential direction of effect for some associations. For example, it is unknown whether the association between lower values alignment and increased burnout is due to perceived misalignment of values contributing to burnout or burnout eroding perception of values alignment. Such interactions are complex and may also be bidirectional. Third, although a standardized instrument was used to evaluate leadership behaviors and all leaders had a at least 5 leader evaluations, the number of evaluations for each leader was limited because of our focus on first-line leaders (who oversee smaller teams of physicians). Fourth, although the instrument used to assess perceived values alignment probes important dimensions, it does not evaluate all elements of individual and organizational values. Fifth, although the values alignment scale demonstrated high internal consistency, we do not have test-retest reliability data. Sixth, although the survey response rate was high, response bias is nonetheless a potential limitation.

## Conclusions

This survey study observed wide variability in perceived personal-organizational values alignment in this large sample of academic physicians. Perceived personal-organizational values alignment was associated with the burnout and professional fulfillment scores of individual physicians. The behavior of the work unit leader had an association with physicians’ perception of values alignment with their organization overall. These findings suggest that people experience their organization through the prism of their local leader. Organizational efforts to improve values alignment should attend to the development of first-line physician leaders.

## Notes

Supplement. **eTable 1.** Participatory Management Leadership Index **eTable 2.** Principal Components Analysis of Leadership Index Items and Personal-Organization Values Alignment Scale Items **eAppendix.** Random Effects Analysis of Variance for Groups Defined by a Specific Leader **eTable 3.** Responses to the Items in the Personal-Organizational Values Alignment Scale **eTable 4.** Multivariable Linear Mixed Model of Factors Associated With Values Alignment Scores
