# Supplementary material for: Assessment of the Association of Leadership Behaviors of Supervising Physicians With Personal-Organizational Values Alignment Among Staff Physicians
Source: JAMA Netw Open. 2021 Feb 9;4(2):e2035622. doi: 10.1001/jamanetworkopen.2020.35622 (PMC7873777; doi:10.1001/jamanetworkopen.2020.35622)
Supplement: Supplement. — eTable 1. Participatory Management Leadership Index eTable 2. Principal Components Analysis of Leadership Index Items and Personal-Organization Values Alignment Scale Items eAppendix. Random Effects Analysis of Variance for Groups Defined by a Specific Leader eTable 3. Responses to the Items in the Personal-Organizational Values Alignment Scale eTable 4. Multivariable Linear Mixed Model of Factors Associated With Values Alignment Scores [file jamanetwopen-e2035622-s001.pdf]

Supplemental Online Content

Shanafelt TD, Wang H, Leonard M, et al. Assessment of the association of leadership behaviors of supervising physicians with personal-organizational values alignment among staff physicians. *JAMA Netw Open*. 2021;4(2):e2035622. doi:10.1001/jamanetworkopen.2020.35622

eTable 1. Participatory Management Leadership Index
eTable 2. Principle Components Analysis of Leadership Index Items and Personal-Organization Values Alignment Scale Items
eAppendix. Random Effects Analysis of Variance for Groups Defined by a Specific Leader
eTable 3. Responses to the Items in the Personal-Organizational Values Alignment Scale
eTable 4. Multivariable Linear Mixed Model of Factors Associated With Values Alignment Scores

This supplemental material has been provided by the authors to give readers additional information about their work.

© 2021 Shanafelt TD et al. *JAMA Network Open.*

eTable 1: Participatory Management Leadership Index (used with permission, copyright Mayo Clinic)[21]

To what extent do you agree or disagree with each of the following statements about (name of immediate supervisor)*?*
Holds career development conversations with me[1]
Empowers me to do my job[1]
Encourages employees to suggest ideas for improvement[1]
Treats me with respect and dignity[1]
Provides helpful feedback and coaching on my performance[1]
Recognizes me for a job well done[1]
Keeps me informed about changes taking place at **Name of organization**[1]
Encourages me to develop my talents and skills[1]
Overall, how satisfied are you with (name of immediate supervisor)[2]

[1] response options: 5 =strongly agree, 4=agree, 3=neither agree nor disagree, 2=disagree, 1=strongly disagree; NA=don't know/not applicable

[2] response options: 5=very satisfied, 4=satisfied, 3=neither satisfied nor dissatisfied, 2=dissatisfied, 1=very dissatisfied

Scoring: sum 1-5 score for each of 9 items to generate a total score (range 9-45). Total scores can be normalized to a 0-10 scale by calculating the mean 1-5 score for the leadership items answered (for those answering at least 7 items), subtracting 1, and multiplying by 2.5.

© 2021 Shanafelt TD et al. *JAMA Network Open.*

eTable 2: Principle Components Analysis of Leadership Index Items and Personal-Organization Values Alignment Scale Items

The results indicate that two components emerge from these 12 items with all 9 items of the leadership scale cluster as one component and the 3 items of the values alignment scale clustering as a separate component.

Standardized loading (pattern matrix) based on correlation matrix:

	PC1	PC2	H2	U2
Leader-career	**0.85**	-0.08	0.66	0.34
Leader-empower	**0.90**	0.02	0.81	0.19
Leader-ideas	**0.85**	0.07	0.78	0.22
Leader-treat	**0.85**	-0.04	0.69	0.31
Leader-feedback	**0.91**	-0.05	0.79	0.21
Leader-recognize	**0.87**	0.04	0.78	0.22
Leader-inform	**0.74**	0.09	0.62	0.38
Leader-talent	**0.89**	0.02	0.81	0.19
Leader-overall	**0.89**	0.00	0.78	0.22
Values-consensus	0.11	**0.83**	0.78	0.22
Values-org	-0.06	**0.92**	0.81	0.19
Values-admin	-0.01	**0.91**	0.81	0.19

	PC1	PC2
SS loadings	6.71	2.41
Proportion Var	0.56	0.20
Cumulative Var	0.56	0.76
Proportion Explained	0.74	0.26
Correlation		
PC1	1.00	0.44
PC2	0.44	1.00

© 2021 Shanafelt TD et al. *JAMA Network Open.*

Screen plot of principle component analysis

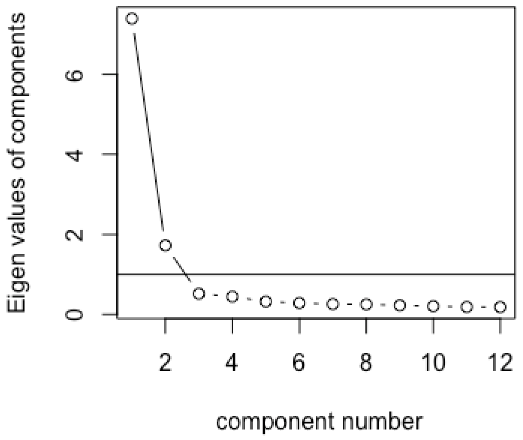

© 2021 Shanafelt TD et al. *JAMA Network Open.*

eAppendix. Random Effects Analysis of Variance for Groups Defined by a Specific Leader

In this cross-sectional study it is difficult to determine the degree to which physicians' reporting of their leaders' behavior accurately measures leaders' actual behavior versus variation in physicians' perception. Analysis of variance with a random effect specified for groups defined by their specific leader shows that this variance component is statistically significant ($p<0.001$). However, the intraclass correlation coefficient is low (ICC = 0.13). Hence, we did observe evidence of clustering of scores by groups defined by leader which is consistent with the hypothesis that actual leadership behaviors account for some portion of variance in the way the physicians they lead rate them. Low intraclass correlation of scores from groups of physicians rating the same leader is not surprising, given the unique dyadic relationships between leaders and each team member they lead." (Reference for text: Gerstner, C. R., & Day, D. V. (1997). Meta-Analytic review of leader–member exchange theory: Correlates and construct issues. Journal of applied psychology, 82(6), 827).

© 2021 Shanafelt TD et al. *JAMA Network Open.*

eTable 3: Responses to the Items in the Personal-Organizational Values Alignment Scale

	N (%)<br>n=868
My input is valued in important administrative decisions	
Not at all true	147 (16.9)
Somewhat true	166 (19.1)
Moderately true	231 (26.6)
Very true	219 (25.2)
Completely true	105 (12.1)
Our organizational goals and values fit well with my goals and values	
Not at all true	64 (7.3)
Somewhat true	186 (21.4)
Moderately true	264 (30.4)
Very true	251 (28.9)
Completely true	103 (11.9)
Administration values my clinical work	
Not at all true	108 (12.4)
Somewhat true	181 (20.9)
Moderately true	238 (27.4)
Very true	234 (27.0)
Completely true	107 (12.3)

© 2021 Shanafelt TD et al. *JAMA Network Open.*

eTable 4: Multivariable Linear Mixed Model of Factors Associated With Values Alignment Scores

	Dependent variable: Values Alignment	
Fixed Effects	Coefficient	Standard Error
Gender (ref=Male)		
Female	-1.25***	0.28
Physician-Leader Gender Concordance (ref=No)		
Yes	0.21	0.28
Age Group (ref=30-39)		
40-49	-0.25	0.35
50-59	-0.09	0.48
>=60	-0.21	0.53
Faculty Track (ref=Clinical Educator)		
Physician Investigator/Biomedical Scientist	0.06	0.34
Other	0.49	0.95
Academic Rank (ref=Instructor)		
Assistant Professor	-0.29	0.52
Associate Professor	0.04	0.61
Professor	-0.60	0.69
% FTE Devoted to Clinical Work (ref=1-20%)		
21%-40%	-0.18	0.48
41%-60%	-1.43**	0.47
61%-80%	-1.11*	0.45
81%-100%	-1.25*	0.50
Hours Worked Per Week (each additional hour)	-0.04***	0.01
Observations	637	
Log Likelihood	-1,595.64	
Akaike Inf. Crit.	3,227.27	
Bayesian Inf. Crit.	3,307.04	

*p<0.05; **p<0.01; ***p<0.001

© 2021 Shanafelt TD et al. *JAMA Network Open.*
